# Harnessing DNA Repair Defects to Augment Immune-Based Therapies in Triple-Negative Breast Cancer

**DOI:** 10.3389/fonc.2021.703802

**Published:** 2021-09-24

**Authors:** Curtis A. Clark, Eddy S. Yang

**Affiliations:** ^1^ Department of Radiation Oncology, University of Alabama at Birmingham (UAB) School of Medicine, Birmingham, AL, United States; ^2^ O’Neal Comprehensive Cancer Center, University of Alabama at Birmingham (UAB) School of Medicine, Birmingham, AL, United States; ^3^ Hugh Kaul Precision Medicine Institute, University of Alabama at Birmingham (UAB) School of Medicine, Birmingham, AL, United States

**Keywords:** TNBC, DNA repair, immunotherapy, PARP inhibition (PARPi), PD-1 - PD-L1 axis, DDR (DNA damage response), breast cancer

## Abstract

Triple-negative breast cancer (TNBC) has poor prognosis with limited treatment options, with little therapeutic progress made during the past several decades. DNA damage response (DDR) associated therapies, including radiation and inhibitors of DDR, demonstrate potential efficacy against TNBC, especially under the guidance of genomic subtype-directed treatment. The tumor immune microenvironment also contributes greatly to TNBC malignancy and response to conventional and targeted therapies. Immunotherapy represents a developing trend in targeted therapies directed against TNBC and strategies combining immunotherapy and modulators of the DDR pathways are being pursued. There is increasing understanding of the potential interplay between DDR pathways and immune-associated signaling. As such, the question of how we treat TNBC regarding novel immuno-molecular strategies is continually evolving. In this review, we explore the current and upcoming treatment options of TNBC in the context of DNA repair mechanisms and immune-based therapies, with a focus on implications of recent genomic analyses and clinical trial findings.

## Introduction

Triple-negative breast cancer (TNBC) is defined by the absence of estrogen and progesterone receptors (ER and PR) and human epidermal growth factor receptor 2 (HER2). This aggressive variant, which accounts for 15-20% of all breast cancers (BC), exhibits a high propensity for early recurrence and metastasis (1, 2). Despite relatively better initial response rates to taxane- and anthracycline-based chemotherapy, durable responses are limited as a result of poorly differentiated tumors with higher rates of acquired resistance to systemic chemotherapy and radiotherapy as compared to other BC subtypes, with median overall survival in metastatic TNBC ranging from 12-18 months (1, 3).

Understanding of specific heterogeneity in TNBC has served as the basis for certain targeted therapies based on particular molecular subtypes previously identified through genomic and transcriptomic profiling (1, 4): The 1) basal-like (BL) subtype exhibits higher rates of BRCA1/2 mutations and expression of DNA damage response (DDR) genes; 2) mesenchymal-like (MES) subtype exhibits stem-like properties, and increased epithelial-mesenchymal transition (EMT), phosphoinositide 3-kinase (PI3K), and Janus kinase (JAK) pathway activation; 3) immunomodulatory (IM) subtype is associated with increased immune checkpoint expression and tumor-infiltrating lymphocytes (TILs); and 4) luminal androgen receptor (LAR) subtype is associated with increased androgen receptor (AR) signaling. For instance, the BL subtype may be potentially more sensitive to alkylating agents, platinums, or poly (ADP-ribose) polymerase (PARP) inhibitors (PARPi’s) as the result of higher rates of BRCA1/2 mutations and DDR deficiency, whereas the MES subtype may be sensitized to protein tyrosine kinase (PTK) and PI3K inhibition given increased activation of these pathways. Likewise, the IM subtype may have increased response to immunotherapy given increased TILs and expression of immune checkpoints, whereas the LAR subtype is potentially more sensitive to AR inhibitors given increased androgen-dependent metabolic activity in this molecular variant of TNBC. However, targeted therapies in TNBC have failed to achieve the remarkable efficacy as observed in other cancers (1, 5).

Pervasive therapeutic resistance in TNBC is another significant challenge, contributing to higher recurrence rates and decreased survival as compared to other BC subtypes (5). Therapeutic resistance in TNBC subtypes occurs through a variety of mechanisms. These include greater antioxidant and autophagy capacity resulting in resistance to radiation- or drug-induced oxidative stress, chemoresistance through upregulation of O-6-methylguanine-DNA methyltransferase (MGMT)-associated activity and mismatch repair (MMR)-deficiency allowing for base mismatched DNA replication (6–8), increased Mcl-1 and Bcl-2-related antiapoptotic activity, and high degree of immunosuppression in part through recruitment of regulatory T cells (Tregs) (9), anti-inflammatory M2 macrophages (10), and increased immune checkpoint (e.g. PD-L1) expression (6, 11–13).

Nevertheless, based on the frequency of DDR deficiency in TNBC, investigation of novel strategies targeting DNA repair defects have generated hope for improved outcomes. PARPi’s aimed at DDR-deficiency in TNBC have been approved for patients with metastatic HER2-negative BC with an inherited BRCA1 or BRCA2 mutation previously treated with chemotherapy (NCT02000622 using Olaparib), and those with deleterious or suspected deleterious germline BRCA-mutated HER2-negative, locally advanced, or metastatic BC (NCT01945775 using Talazoparib). However, these have restricted application and demonstrate modest albeit intriguing clinical benefit at present (14–16). A recent report also suggested benefit of PARPi in patients with metastatic breast cancer beyond germline BRCA1/2 mutations (NCT02032823 using Olaparib) (17).

Another promising therapy for TNBC exploits the immune system. Given the immunogenic characteristics of TNBC, immunotherapy represents a promising treatment strategy for this aggressive breast cancer with few efficacious systemic options at present. The most successful immunotherapeutic agents to date consist of immune checkpoint inhibitors (ICIs), which block immune co-inhibitory receptors, such as cytotoxic T-lymphocyte antigen 4 (CTLA-4) and programmed cell death protein 1 (PD-1), or associated ligands such as programmed cell death ligand 1 (PD-L1), to dis-inhibit TILs and permit tumor-specific cytotoxicity. However, highly immunosuppressive tumor microenvironment (TME) competes with ICI-enhanced anti-tumor immunity and significantly contribute to inconsistent clinical responses. Immunotherapies, particularly combination strategies, represent a refined approach to treating cancers with immune modulating DDR defects, high tumor mutational burden (TMB), and intact anti-tumor immunity, which are all characteristics frequently observed in TNBC. Tumors with intact interferon-gamma (IFN-γ) pathway signaling, robust TILs, increased immune co-inhibitory receptor expression, and high TMB/neoantigen expression have been shown to respond better to immune checkpoint inhibition than weakly immunogenic tumors with inadequately established anti-tumor immunity (18). As such, TNBC typically exhibits properties favorable to immunotherapy response, including increased TILs (19), which correlates with improved outcomes in early-stage TNBC (20), higher PD-L1 expression as compared to hormone receptor positive BC (12, 13), and increased TMB giving rise to tumor neoantigen-specific T cells (2, 18, 21, 22). The PD-L1 mAb, Atezolizumab, is an FDA-approved ICI for patients with PD-L1 positive, unresectable, locally advanced, or metastatic TNBC (NCT02425891). However, ICI monotherapy efficacy is limited in TNBC, with response rates in the 5-25% range (23), suggesting coexisting immunosuppressive or tumorigenic factors at play that overwhelm or subvert ICI-enhanced anti-tumor immunity. Thus, improved strategies that augment the immunotherapeutic potential of ICIs are needed.

Given the immunosuppressive phenotype associated with TNBC (6, 10, 11, 13), it is feasible that innate and acquired immune resistance mechanisms have in part curbed robust outcomes using various approved inhibitors in TNBC patients. Furthermore, DDR-targeting therapies have been shown to augment anti-tumor immunity as well as immune checkpoint signaling (24–27), potentially opening the door to combination immunotherapy in TNBC patients with DDR-deficiency and inadequate or exhausted TILs.

This review summarizes the promising role of DNA repair deficiency as a surrogate biomarker to guide the use of ICI therapy in TNBC, discusses underlying mechanisms that link DDR signaling to anti-tumor immunity, and outlines the emerging evidence describing the relationship and potential cooperative therapeutic potential between DDR-pathway targeting agents and immunotherapy.

## 1 DNA Damage Repair and Associated Defects in TNBC

**Figure 1:**
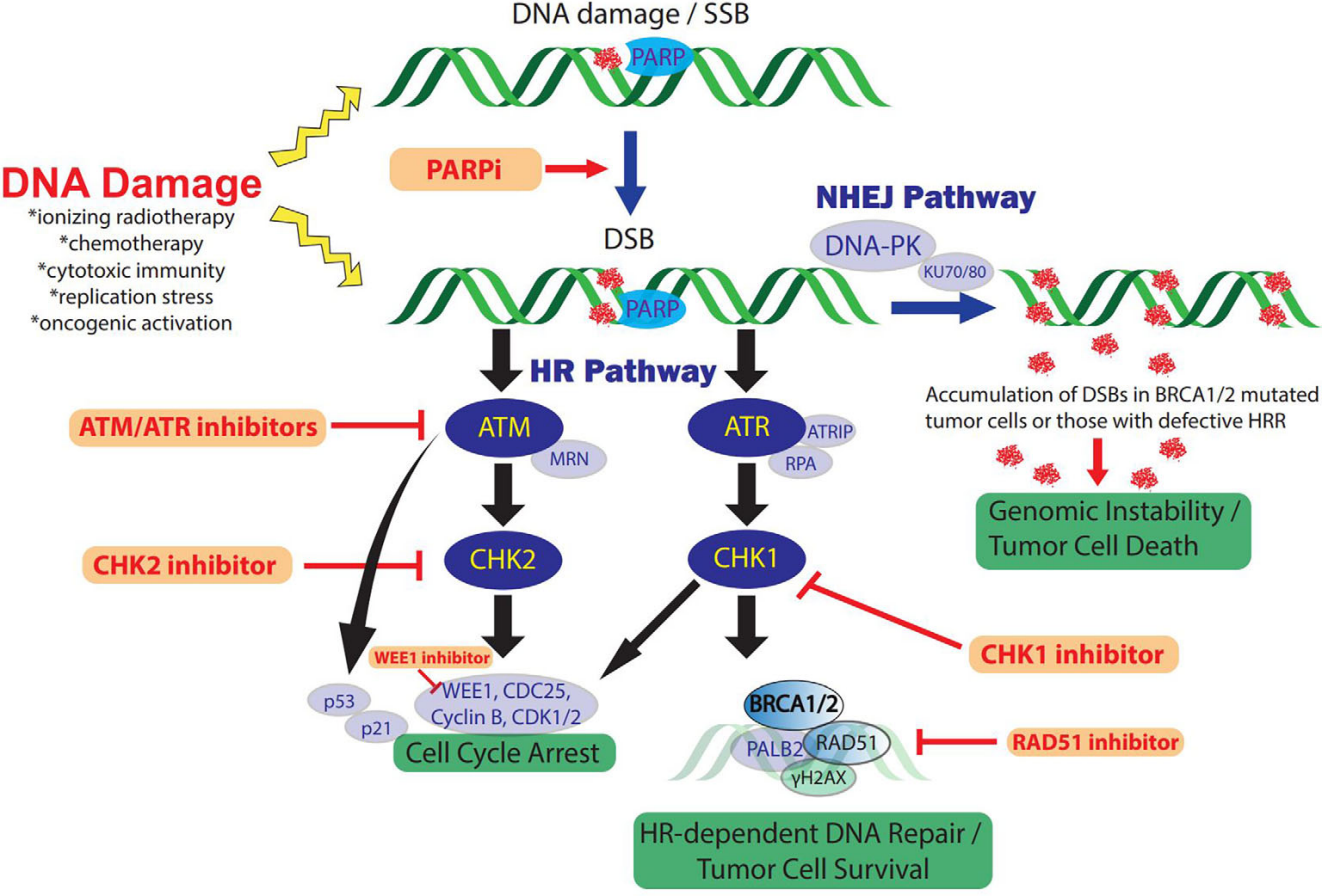
**The DDR and therapeutic strategies in TNBC**. DNA-damaging therapies or endogenous replication dysfunction result in SSBs and DSBs which activate the DDR and repair signaling pathways. Distinct DSB DDR signaling pathway initiation depends on the type of DNA damage and is mediated by three central DDR kinases: DNA-PK, ATM, and ATR. In addition, PARP enzymes play a key role in DDR and facilitate SSB repair efficiency and functions in DSB repair *via* HRR and NHEJ pathways. The ATM and ATR pathways cross-talk extensively and only key intersections are highlighted here for pragmatic purposes. ATM/CHK2 signaling induces cell cycle arrest, preventing cell cycle progression in tumor cells with DNA damage. In addition, ATR/CHK1/WEE1 signaling initiates DNA DSB repair by inducing checkpoints and activating key components of HRR, including BRCA1/2 activity. Alternatively, DSB repair occurs through NHEJ *via* DNA-PKcs recruitment. Inhibition of PARP to treat TNBC with defects in HRR such as BRCA1/2 mutations, induces DSBs from unrepaired SSBs *via* PARP trapping and collapsed replication forks. Accumulated PARPi-induced DNA damage cannot be effectively repaired due to the HRR deficiency, resulting in genomic instability and cell cycle arrest. In addition, loss of function or inhibitors (red) against other key mediators of HRR also constrain NHEJ dependence which can be overwhelmed in the setting of concomitant PARPi *via* accumulation of DSB and genomic instability. RAD51 inhibition also suppresses HRR and sensitizes TNBC to PARPi. ATM/ATR/CHK1/WEE1 inhibition increases DSBs and impairs cell cycle arrest checkpoints and DNA damage repair, ultimately resulting in tumor cell death. DDR, DNA damage response; SSB, single-strand breaks; DSB, double-stranded breaks; DNA-PK, DNA-dependent protein kinase; ATM, ataxia telangiectasia-mutated; ATR, ataxia telangiectasia and Rad3-related protein; NHEJ, non-homologous end-joining; HR(R), homologous recombination repair; PARP, poly (ADP-ribose) polymerase; PARPi, PARP inhibitor/inhibition; MRN complex, Mre11, Rad50, Nbs1; ATRIP, ATR-interacting protein; RPA, replication protein A.

Cells routinely undergo DNA damage as the result of cytotoxic stress. In normal physiology, mechanisms of DNA damage detection and repair are critical to preserve genomic integrity and thwart malignancy when DNA damage exceeds the cellular repair threshold. DDR accomplishes this by arresting proliferation and facilitating removal of damaged cells through activation of senescence or apoptosis. As such, defects in DDR genes permit mutations and chromosome rearrangements advantageous for tumor initiation and progression. In TNBC, with alkylating chemotherapies and radiation as major components of therapy, aberrant DDR signaling represents a dominant mechanism of tumorigenesis and treatment resistance, while also yielding potential therapeutic synergies with platinum chemotherapies or targeted therapies. An overview of the DNA damage response and repair pathways is detailed below and shown in **Figure 1**.

### 1.1 DNA Damage Response and Repair Pathways

Depending on the mechanism of DNA damage and lesion formation, DDR is achieved by various pathways (28, 29). DNA single-strand break (SSB) damage is remedied by three main pathways: base excision repair (BER), nucleotide excision repair (NER), and mismatch-repair (MMR). More severe DNA double-strand breaks (DSBs) are restored by two additional pathways: homologous recombination (HR) and non-homologous end joining (NHEJ) (28, 29). Ataxia telangiectasia mutated (ATM), ATM- and RAD3-related (ATR), and DNA-dependent protein kinase (DNA-PK), in cooperation with many other mediators, act as core sensors that regulate DDR and coordinate DSB signaling. ATM and ATR protein kinases, operating together *via* downstream targets Checkpoint Kinase 1 (CHK1) and Checkpoint Kinase 2 (CHK2), respectively, play a vital role in DDR signaling by maintaining replication fork stability and the regulation of cell cycle control checkpoints (30). Additionally, DNA-PK activity is required for NHEJ, and a WEE1 nuclear kinase regulates mitotic entry and nucleotide reservoirs during DNA damage response (30, 31). Loss of function mutations in crucial genes involved in DDR, such as BRCA1/2, BRD4, PTEN or TP53, are associated with cancer-prone cellular behavior and malignant phenotypes. Consequently, failure in DDR results in impaired removal of genome mutations, accumulation of DNA damage and increases the risk of oncogenesis (32). In reflexive response to DDR deficiency, tumor cells activate alternate DDR pathways, thereby counteracting sensitivity to genomic insult by preventing lethal cytotoxic stress and perpetuating oncogenesis, which is altogether a problematic mechanism of resistance to DNA-damaging cancer treatments. As a result of tumor cells often harboring oncogenic defects in DDR pathways and therefore increased dependence on alternate DDR mechanisms to survive, there is increased susceptibility to DDR inhibition and subsequent accumulation of lethal levels of DNA damage as compared to normal cells (5). These DDR defects will cause accumulation of significant DNA alterations that not only can facilitate oncogenesis, but it is becoming ever more evident that these changes can modify the TME and inflammatory cascade (33).

Therapeutic targeting of DDR pathways in TNBC is therefore a promising strategy given the propensity for therapeutic resistance and DDR deficiency. Furthermore, increasing evidence demonstrates a link between DDR deficiency and activation of anti-tumor immunity, and we will discuss the potential for combined approaches targeting genomic and immunologic aspects of TNBC tumorigenesis later in this review.

### 1.2 The Role of PARP in DNA Damage Repair

DNA base damage, such as base loss or SSBs, results in BER. Poly (ADP-ribose) polymerases (PARP1/2) are important DNA-damage sensors and regulators of BER-mediated SSB repair as well as other DDR pathways (25). These enzymes bind *via* zinc finger domains to SSBs *via* co-factor nicotinamide (β-NAD+) and catalyze the synthesis of PARP chains (auto-poly (ADP-ribosylation), resulting in activation of intracellular signaling pathways that enable chromatin remodeling and recruitment of DDR-related protein machinery, thereby preventing accumulation of SSBs (34–36). In the setting of a HR deficiency, PARP inhibition disrupts efficient DNA damage repair resulting in increased genomic instability, stalled replication fork extension and lethal DSBs.

### 1.3 Synthetic Lethality and Clinical Utility of PARP Inhibitors in TNBC

Clinical use of PARP inhibitors (PARPi’s) is an important example of DDR-specific targeting of HR defective cancers (14, 37). PARP1 inhibition can cause the accumulation of SSBs and subsequent DSBs. HR is required for DSB repair, and HR-deficiency is a typical pathological feature of the BRCA1/2-mutated tumor and enables enhanced response to PARP1 inhibition due to synthetic lethality. PARPi’s in cells deficient in HR are unable to effectively undergo DDR, whereas PARPi is well-tolerated by normal cells. As such, this effect of PARPi is more likely observed in tumor cells with a BRCA-deficient background or tumors with underlying deficiencies in HR (38). Tumors cells with intact HR signaling can overcome PARP inhibition preferentially by HR rather than NHEJ (34), whereas cells with HR deficiency (HRD), including those with mutations in BRCA1/2, BRD4, and PTEN, demonstrate sensitivity to PARP inhibition resulting in cell death (35, 36, 38). PARPi therefore represents a synthetic lethal therapeutic approach for the treatment of cancers with compromised ability to repair double-strand DNA breaks by HR, including those with defects in BRCA1/2 (17, 34, 38). Numerous PARPi’s have been developed, including Olaparib, Rucaparib, Niraparib, Talazoparib, and Veliparib, which are primarily applied in cancer patients with BRCA1/2 mutations (14, 16, 17, 39). Altogether these studies demonstrate that sensitivity of HRD-TNBC tumor cells to DNA-damaging agents may be the direct result of associated defective DDR mechanisms.

Although the greatest efficacy of PARPi has been observed in tumors with BRCA1/2 mutations, consensus is that synthetic lethality insufficiently explains PARPi-related anti-tumor activity. For example, the degree of PARP catalytic inhibition is poorly correlated to PARPi-induced cell-killing in HRD cells (40). In addition, PARPi induces cytotoxicity to a greater extent than PARP depletion, suggesting associated mechanisms contribute to anti-tumor activity (40, 41). In addition, loss of other tumor suppressor DDR proteins, many of which are involved in HR, such as RAD51, ATR, ATM, CHK1, CHK2, and partner and localizer of BRCA2 (PALB2), also have been shown to permit sensitization to PARPi (35, 40). HRD has also been shown to regulate sensitivity to alkylating chemotherapy in some TNBC patients (42), whereas the ATR-CHK1 cascade may conversely regulate resistance to chemotherapy by preventing replication stress. Further emphasizing the role of these accessory molecules in preventing susceptibility to DNA repair targeting, it was reported that ATR inhibition was effective in sensitizing both HR-proficient and deficient TNBC cells to ionizing radiation therapy (43). These results suggested that PARPi might be a useful therapeutic strategy not only for the treatment of BRCA-mutated tumors but also for the treatment of a wider range of non-BRCA-mutated tumors that are inherently HRD or ‘BRCAness/HRDness’ (15, 34).

In the context of TNBC, there is a higher degree of ‘BRCAness’ as compared to other breast cancer subtypes (1, 5), As such, PARPi’s have demonstrated the potential for increased therapeutic efficacy in TNBC patients with HRD/BRCAness, due to increased accumulation of DSBs and incidence of synthetic lethality (35, 36). Olaparib, an orally active PARPi, was the first to be shown to induce synthetic lethality in BRCA-deficient cells and exhibit potential clinical benefit in patients with TNBC having BRCA deficiency. At present, Olaparib and Talazoparib are FDA-approved as single-agent regimens for previously chemotherapy-treated, HER2 negative, metastatic breast cancers with germline BRCA mutations, which primarily constitutes TNBC. In addition to exploiting BRCAness in TNBC, PARPi’s have been shown to radiosensitize breast cancer cells through DDR inhibition, and clinical trials in breast cancer patients explore their potential to enhance the response of cancers to ionizing radiation (44).

Use of PARPi’s in TNBC is supported by findings from the phase III OlympiAD trial of metastatic breast cancer (16), which demonstrated an approximately two-fold increase in response rate (59.5%), increased median progression-free survival (PFS; 7.0 months), and less toxicity as compared to conventional chemotherapy in patients with metastatic HER2-negative breast cancer with germline BRCA1/2 mutations treated with Olaparib (NCT02000622). A phase I study of Talazoparib demonstrated promising efficacy and safety profiles in advanced cancers with deleterious BRCA1/2 mutations including breast cancer (NCT01945775). A phase III trial EMBRACA comparing Talazoparib versus physician’s choice standard of care in metastatic TNBC revealed significant benefit of Talazoparib with better PFS and objective response rates (ORRs) (14, 39). Other PARPi’s, including Veliparib, and Rucaparib, have been investigated in metastatic breast cancer. Trials of veliparib in combination with alkylating agents are currently underway for advanced or metastatic TNBC (45–47). In early TNBC, the phase III OlympiAD trial (NCT02032823) is currently ongoing to evaluate adjuvant Olaparib monotherapy after standard neoadjuvant therapy in high-risk TNBC with germline BRCA1/2 mutations. Another phase I trial of neoadjuvant monotherapy with the novel PARPi Niraparib is underway (NCT03329937). The phase II/III PARTNER trial of neoadjuvant Olaparib in combination with carboplatin followed by the standard chemotherapy is under investigation in patients with TNBC and/or germline BRCA mutations (NCT03150576). The I-SPY 2 trial, which evaluated neoadjuvant Veliparib and carboplatin in addition to the standard chemotherapy in patients with high-risk breast cancer and TNBC, demonstrated significant benefit from this combination therapy (pathologic complete response (pCR) rates: 52% *vs* 24%) (48). In a recent biomarker analysis of the I-SPY2, a BRCA1ness gene signature was identified as a significant predictive biomarker of response to neoadjuvant combination Veliparib and carboplatin (49). Conversely, a phase II neoadjuvant trial in high-risk, residual TNBC after standard neoadjuvant chemotherapy failed to show a significant therapeutic benefit from the combination of low-dose Rucaparib and cisplatin compared with cisplatin alone; although the lack of benefit may be due to a therapeutically insufficient rucaparib dose (NCT01074970). Altogether, additional studies are required to elucidate the clinical benefit of PARPi addition to platinum-based chemotherapy in TNBC as platinum alone demonstrates efficacy either as monotherapy or in combination (50, 51). This also further emphasizes the need to identify additional therapies that sidestep resistance to therapeutic targeting of DDR deficiency.

Other strategies to exploit HR include inducing a synthetic lethality by generating a BRCAness phenotype. These promising preclinical studies include combinations with inhibitors of EGFR, PI3K, BET, and others (52–54). We recently reported promising results of a clinical trial with lapatinib and veliparib in non-BRCA1/2 mutated TNBC based on an induced DNA repair deficiency with EGFR inhibition (NCT02158507) (55).

### 1.4 Role of MMR and NHEJ in TNBC

In TNBC, defective MMR allows DNA replication with mismatched bases and facilitates resistance to anti-metabolites and alkylating agents. Whole-genome sequencing studies have shown that approximately 5-7% of TNBC patients are MMR-deficient (6, 8), as compared to approximately 2% in other breast cancers. Furthermore, MMR status corresponds to PD-L1 expression and CD8^+^ T cells in the TNBC TME versus poor correlation in other subsets of breast cancers. Altogether, these findings indicate the immunotherapeutic efficacy potential in TNBC with MMR deficiency (6). In the context of immunotherapy, MMR deficiency not only has the potential to elicit more tumor antigens and improved immune checkpoint inhibitor response (56). The TMB/neoantigen/IFN-γ pathway is a well understood cancer pathway that results in PD-L1 upregulation, supported by the finding that even partial loss of MMR significantly correlates with increased PD-L1 expression suggesting a therapeutic vulnerability in HRD TNBC (6). Mounting evidence indicates that DDR defects are also important in driving sensitivity and response to ICI. Given that MMR deficient (dMMR) tumors harbor a large number of mutations, which are associated with high neoantigen load and T-cell infiltration, it is not surprising that dMMR tumors can respond well to immune checkpoint blockade. Indeed in many cancers, MMR deficiency predicts efficacy of anti-PD-L1 (Pembrolizumab), and microsatellite instability (MSI)/dMMR is a validated DDR defect biomarker for predicting response to ICI therapy (56). Furthermore, Pembrolizumab is FDA-approved for solid tumors based solely on the presence of MSI-status as a biomarker, irrespective of cancer type (56). Although MSI or dMMR rarely appears in breast cancer (57), as we will discuss further, the therapeutic potential in combining with immune-stimulating DNA repair inhibitors remains intriguing.

The NHEJ signaling pathway is an important mediator of DSB repair. The Ku70-Ku80 heterodimer and DNA-dependent protein kinase catalytic subunit (DNA-PKcs) initiate NHEJ, and these complexes have been shown to be regulated by EGFR amplification and/or p53 mutation-induced overexpression of long non-coding RNA in the NHEJ pathway 1 (LINP1), resulting in NHEJ-mediated chemo- and radiation resistance (58, 59). Doxycycline, an FDA-approved agent that can inhibit DNA-PK, has been shown to reduce DNA-PKcs expression and sensitize breast cancer cells to radiation (60). Although more investigation is necessary, these findings suggest that targeting of NHEJ-related mediators may be useful in TNBC, particularly those with EGFR, p53 and/or DDR-associated mutations resistant to DNA-damaging agents.

### 1.5 Role of Radiation Therapy in DNA Damage Signaling and Immune Strategies

Most breast cancer patients receive ionizing radiotherapy (RT) as part of their treatment to improve locoregional control by inducing tumor cell death predominately through the generation of DSBs, which in turn can elicit either protective anti-tumor immune responses or immunosuppression (61). Unfortunately, positive immune effects of radiation are often insufficient to shift the balance of the immunosuppressive TME to achieve tumor rejection, especially in the absence of targeted immunotherapy. Combining immune checkpoint blockade with radiotherapy has thus emerged as an exciting dual modality treatment approach for a myriad of cancer types, although clinical outcomes are highly variable.

#### 1.5.1 Impact of Radiation Therapy on Anti-Tumor Immunity and Immunosuppression

RT-enhanced tumor immunogenicity can occur through multiple mechanisms, including increased antigen availability, inflammatory cell infiltration into tumors, and increased priming and exposure of phagocytic and cytotoxic cells to tumor-associated antigens (62). Specifically, RT can up-regulate FAS (death receptor) and MHC class I on tumor cell surfaces, alter the repertoire of peptides presented by MHC, cause translocation of calreticulin to tumor cell surfaces resulting in enhanced antigen uptake by antigen presenting cells, and induce release of HMGB1 from dying tumor cells. These actions induced by RT can result in dendritic cell maturation and chemokine and cytokine secretion that promotes TIL trafficking (62, 63). Furthermore, RT-induced DSBs and subsequent ATM activation has been shown to regulate pattern recognition receptors that activate interferon and innate immune system signaling (64, 65). Local and systemic immune effects include RT-induced alteration of chemokine signaling, cell trafficking, and secondary immune system activation *via* dendritic cell cross-presentation of tumor-derived antigens to T cells (63, 66).

The link between radiation and both local and systemic anti-tumor immune effects has been investigated in many preclinical and clinical studies (61, 63, 65). It has been reported that immune-related therapeutic effects of locally ablative RT require intact immunity, type I interferon production and infiltration of CD8^+^ T cells (67), highlighting the importance of functional anti-tumor immunity in the current era of radio-immunotherapy. However, RT and the resultant tumor cell death can also potentiate immunosuppressive TMEs, as studies have shown that radiation can induce lymphopenia, immune dysfunction through release of immunosuppressive cytokines (TGF-β, IL-10) and chemokines, and induction of immunosuppressive immune cells including myeloid-derived suppressor cells (MDSCs), M2 tumor-associated macrophages (TAMs), T regulatory cells (Tregs), which can all result in immune escape and tumor progression (62, 66).

Importantly, radiation can further induce immunosuppression and adaptive immune resistance *via* upregulation of checkpoint pathways, including PD-L1 expression on the tumor cell surface (65, 68). Although the neoantigen-T cell activation-IFN-γ-STAT1/3-IRF1 pathway of PD-L1 induction has historically been viewed as the chief mediator of this adaptive immune resistance, recent work has implicated DNA damage and repair signaling in the regulation of tumor PD-L1, including through radiation-mediated DSBs and cytosolic DNA sensing. DNA damage dependent PD-L1 expression is upregulated by ATM/ATR/CHK1 kinase activities and the cyclic-GMP-AMP ((cGAMP) synthase (cGAS))/stimulator of interferon genes (STING)-dependent pathway. Altogether, tumor cell PD-L1 expression is controlled by the STAT-IRF pathway which is regulated by distinct DNA damage mechanisms: 1) DSB-induced ATM/ATR/CHK1 kinase activities, 2) DDR deficiency/high MSI/increased TMB resulting in neoantigen-induced T cell activation and IFN-γ production, and 3) cytosolic DNA fragments that induce the cGAS/STING pathway resulting in type I interferon activity (68).

RT induced PD-L1 expression *via* activation of the cytosolic DNA sensing cGAS/STING pathway represents a novel mechanism of adaptive immune resistance. The cGAS/STING, with subsequent type I interferon production, is a fundamental immunostimulatory pathway in antimicrobial innate immunity (64), and has been found to mediate the TME and immune milieu, including immune surveillance, dendritic cell function and CD8^+^ T cell function (69). Interestingly, STING-activity is also upregulated in the setting of DDR deficiencies including BRCA1/2 and ATM mutant tumor cells (69). This STING-dependent interferon signaling can initially facilitate immune activation; however chronic STING pathway activation and/or IFN-γ signaling can ultimately lead to T cell exhaustion *via* PD-L1-dependent resistance to anti-tumor immunity (70), potentiating cancer immune escape.

RT-induced DSBs and subsequent ATM/ATR/CHK1 kinase activities have also been implicated in upregulation of tumor PD-L1 expression through direct STAT1/3-IRF1 activation (26, 66, 68), independent of neoantigen production. Consistent with this, Ku or BRCA2 defects were found to augment RT-induced PD-L1 expression (26, 68), and ATR inhibition reduced upregulation of PD-L1 following RT. Interestingly, ATR inhibition potentiated CD8^+^ T cell activity and reduced RT-induced T cell exhaustion (71). Furthermore, RT-induced interferon signaling has been shown to be dependent on cGAS/STING pathway activation (65). This evidence suggests a novel PD-L1-dependent, immunosuppressive consequence of DNA damaging therapies (e.g., chemotherapy, RT, DDR inhibitors). In relation to immune-activating properties of RT, the disadvantageous PD-L1 induction following RT represents a therapeutic opportunity with combination ICI therapy that would result in more durable clinical responses.

#### 1.5.2 Clinical Application of Radio-Immunotherapy Combinations in TNBC

Observations in patients receiving ICI and RT have demonstrated the potential for improved clinical responses in various primary and metastatic malignancies, and numerous clinical trials are underway investigating potential synergy. Clinical trials evaluating patients with metastatic cancer have established that RT combined with ICI is safe and well-tolerated, and can potentially halt tumor growth by stimulating anti-tumor immunity (61, 66). In TNBC, a phase II trial evaluated PD-L1 inhibition (Pembrolizumab) plus RT in patients with metastatic TNBC patients who were unselected for PD-L1 expression. In this study, the ORR for the entire cohort was 17.6% (3 of 17 patients; 95% CI: 4.7%-44.2%), with 3 complete responses of tumors outside of the irradiated portal (72). The context dependence of the robust synergistic effects of RT and ICI are potentially consistent with fluctuating immune-tolerance and suppression mechanisms, particularly in the locally advanced or metastatic setting. This altogether highlights the need for larger clinical trials assessing predictive biomarkers and investigation of additional targeted strategies. For instance, the phase I RADIOPARP trial is investigating PARP1 inhibitors (Olaparib) in combination with RT in the setting of advanced or metastatic TNBCs (73). Neoadjuvant Veliparib combined with RT is under exploration in a phase I study for node-positive, residual BC following neoadjuvant chemotherapy (NCT01618357). These and additional studies are needed to optimize radiotherapy modulation of DDR-dependent immune augmentation and anti-tumor immunity in the context of ICIs.

## 2 Cancer Immunology in TNBC

Immune evasion is a hallmark of cancer that is the result of a complex TME consisting of stroma, myeloid and lymphoid immune cells, dysregulated lymphovascular networks. The interaction of these components often plays roles in tumorigenesis, tumor heterogeneity, and adaptive and therapeutic resistance. Central to immune-mediated tumor rejection are TILs, a heterogeneous population that contributes to competing innate and adaptive anti-tumor and immunosuppressive effects. TILs, including CD8^+^ T and NK cells that are central to anti-tumor immunity in breast cancer, have prognostic significance even in systemically untreated early TNBC, suggesting that the presence of TILs may delineate candidates most likely to benefit from adjuvant chemotherapy or immunotherapy (74).

The TME of TNBC is often abundant in TILs because of inherent genomic instability and high mutational burden. As the result of these genetic and epigenetic aberrations, anti-tumor TILs engage in immune-mediated tumor cell killing and tumor cell immunoediting, often times resulting in subset(s) of immune resistant tumor cells (2, 75, 76). In metastatic TNBC, response rate and overall survival after Atezolizumab significantly correlated with TIL levels (77). However, in early TNBC, retrospective studies demonstrated significantly worse survival outcomes in patients harboring high PD-L1 expression and a low number of TILs or a high ratio of PD-L1/CD8 expression (12, 78), suggesting that TIL alone is not indicative of the immune activity or suppression status. Consistent with this, immunologic signatures associated with higher mutational burden positively correlated with higher TILs and a more favorable prognosis (12), suggesting antigen-specific anti-tumor TILs likely play a significant role in coordinating the functional state of anti-tumor immunity and response to immunotherapy (12). Furthermore, TILs are shown to be a robust predictive biomarker of long-term survival in TNBC patients treated with neoadjuvant therapies and to facilitate improved response to cytotoxic agents (79–81). However, effective anti-tumor TIL activity is frequently hindered by immunosuppressive immune cells types such as regulatory Tregs and MDSCs, which are also typically found in higher concentrations in TNBCs. Nevertheless, compared with other BC subtypes, TNBC exhibits a higher degree of lymphocytic infiltration (19), and studies to date indicate that TILs are useful biomarkers and potential therapeutic targets in TNBC.

### 2.1 Immune Co-Inhibitory Pathways in TNBC

Upon activation, T cells begin to express co-inhibitory cell surface receptors that control T cell function, such as CTLA-4 and PD-1. The balance between co-stimulatory and co-inhibitory signals is crucial for cytotoxic T cell activation and immunologic tolerance. Tumors can exploit this balance to escape T cell-mediated, tumor antigen-specific immunity. Importantly, therapeutically targeting these co-inhibitory pathways with immune checkpoint inhibitors (ICIs) is capable of unleashing anti-tumor activity (78, 82). In TNBC, immune co-inhibitory signaling is often upregulated and is associated with immunosuppression, MMR-status and mutational burden, chemoresistance and overall poor prognosis (6, 11, 12).

CTLA-4, an immune checkpoint constitutively expressed on Tregs and transiently upregulated on activated T cells, inhibits early T cell priming by antigen-presenting cells (APCs) in the lymph nodes (83). The expression of CTLA-4 on Treg cells competitively blocks the binding of CD28 to the CD80/86 proteins on APCs, thereby turning off T cell activation (82). CTLA-4 blockade has demonstrated efficacy in anti-tumor immune activity in some cancers by allowing tumor antigen-specific T cell stimulation. CTLA-4 ICI has demonstrated durable response in a small subset of patients with metastatic TNBC (84, 85), and CTLA-4 mAbs, including Ipilimumab and Tremelimumab, are being investigated with the PD-1-axis immunotherapies Durvalumab and Nivolumab, respectively, for TNBC.

PD-1, another immune checkpoint, is widely expressed on activated anti-tumor immune cells, including T and natural killer (NK) cells, and APCs, and yields inhibitory signals through binding of its two ligands, namely PD-L1 and PD-L2 (86). PD-L1 is highly inducible and expressed on many cancers in response to anti-tumor immune activity and inhibits PD-1^+^ tumor antigen-specific CD8^+^ T cells (87), representing a key mechanism underlying cancer adaptive immune resistance. Correlation between TILs and PD-1/PD-L1 expression is well studied, as tumor-associated inflammation promotes adaptive upregulation of immunosuppressive PD-L1 expression in response to anti-tumor immune cell production of IFN-γ and tumor cell STING pathway activation (22, 88). Blockade of PD-1/PD-L1 interaction is capable of restoring T cell function and tumor elimination. However, in breast and other cancer cell types, meaningful response is inconsistent as a result of reduced or heterogeneous PD-L1 expression, immunosuppressive mechanisms, impaired immune cell function and trafficking of TILs (87, 89), resulting in paradoxical PD-L1^+^ “non-responders” and PD-L1^low/null^ “responders”.

There is compelling evidence that resistance to DNA-damaging agents may play a meaningful role in immunotherapy outcomes. For example, defects in BRCA1/2 correlates to higher levels of PD-L1 expression (90, 91). In addition to inactivation of PD-1^+^ anti-tumor immune cells, tumor PD-L1 also mediates diverse cell-intrinsic functions that increase cancer virulence, including mTORC1 promotion and autophagy suppression (92–94), that can not only alter immune infiltrates and enable immune escape (94–98), but may also play a role in response to DNA-damaging therapies. Indeed, it has been shown that tumor-intrinsic PD-L1 can regulate IFN-γ-induced apoptosis, DDR, RT and chemotherapy resistance, and effects on Ras/Mek/ERK, PI3K/AKT, JAK/STAT (94, 99–101); which, altogether may create treatment-exploitable immune signaling effects.

**Figure 2:**
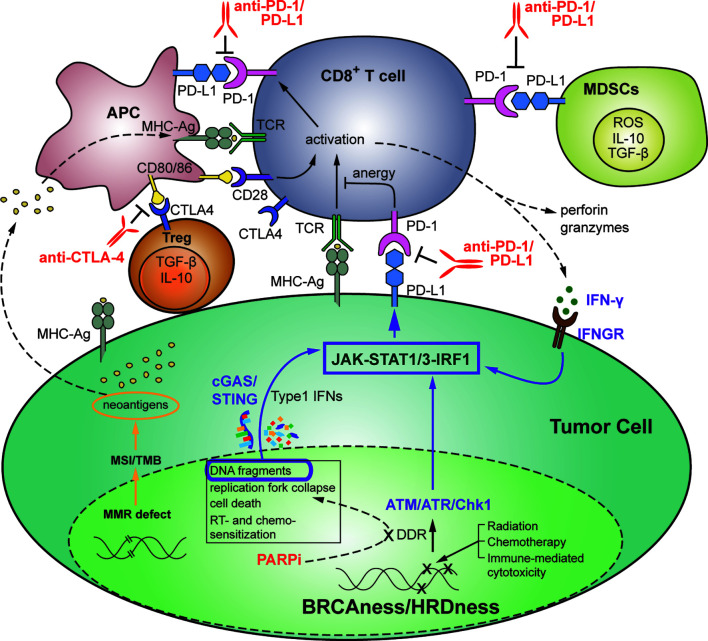
**Therapeutic strategies targeting the interplay between DDR and anti-tumor immunity in the setting of HR-deficient triple-negative breast cancer.** DNA damage affects the balance between tumor progression and immune surveillance. Genomic stress induced by DNA-damaging treatments or by defects in DDR or MMR results in accumulation of chromosomal abnormalities, higher TMB, oncogene activation and tumorigenesis, as well as immune recognition, activation of immunostimulatory genes, and increased TILs including anti-tumor immune cells (CD8^+^ T cells, APCs, CD4^+^ T cells, and NK cells). Immunosuppressive immune cells such as CD4^+^ Tregs, MDSCs and M2 macrophages can also be increased. Targeting of SSB and DSB repair with inhibitors of DDR, including PARPi’s, in the setting of TNBC with BRCA or HR-related mutations (BRCAness/HRDness) can, in addition to inducing synthetic lethality, increase generation of cytosolic DNA fragments. This results in activation of the immunomodulatory cGAS/STING pathway that promotes anti-tumor immunity through activation of T and NK cells, neoantigen recognition, and increased PD-L1 expression *via* the JAK-STAT1/3-IRF1 pathway. Anti-tumor immunity can further contribute to tumor PD-L1 expression *via* IFN-γ-dependent activation of IRF1. Tumor and immune cell expressed PD-L1 subsequently suppresses PD-1^+^ cytotoxic anti-tumor immune cells *via* inhibitory binding. Thus, DNA-damage induced anti-tumor immune response is often overwhelmed by coexisting immunosuppressive factors, and the balance in favor of anti-tumor immune rejection can be mediated by ICIs such as anti-PD-1/PD-L1 and anti-CTLA-4 mAbs. APC, antigen presenting cells; ICI, immune checkpoint inhibitor; mAb, monoclonal antibody; MDSC, myeloid-derived suppressor cells; MMR, mismatch repair; NK, natural killer; TIL, tumor-infiltrating lymphocyte; TMB, tumor mutational burden; Treg, T regulatory cell.

The interaction of these pathways to modulate the immune system is depicted in **Figure 2**.

### 2.2 Role of Immune Checkpoint Inhibition in TNBC

ICIs, including monoclonal antibodies against PD-1 (Pembrolizumab, Nivolumab), PD-L1 (Atezolizumab, Durvalumab, Avelumab), and CTLA-4 (Ipilimumab), have generated durable responses across many tumor types (102). Clinical studies using PD-1/PD-L1 mAb therapies have demonstrated promise in patients with PD-L1 positive TNBC, and Atezolizumab is FDA-approved for patients with PD-L1^+^, unresectable, locally advanced, or metastatic TNBC (NCT02425891). Studies of Atezolizumab in advanced solid cancers, including heavily pretreated TNBC, demonstrate limited but impressive outcomes, as only 10% of patients experienced clinically meaningful response, with 100% survival rate at 2 years in these responders, and median PFS of PD-L1^+^ TNBC patients treated with Atezolizumab plus nab-PTX was significantly increased by 50% (7.5 months *vs.* 5 months) (103). KEYNOTE-012 and KEYNOTE-086 studies demonstrated durable response with Pembrolizumab in approximately 20% of patients with metastatic TNBC (104, 105). Patients with positive PD-L1 expression treated with first-line Pembrolizumab showed a higher response rate than patients with any level of PD-L1 expression. Using Avelumab in patients with heavily pre-treated metastatic TNBC, the phase I JAVELIN trial demonstrated promising efficacy outcomes with a 31% control rate, and PD-L1 expression correlated with response (106). Currently, a phase II trial of Pembrolizumab as monotherapy for BRCA-mutated breast cancer is underway (NCT03025035). These studies using PD-1-axis inhibitors demonstrate therapeutic benefit in some patients, but future studies are required to address inconsistent responses, better define the therapeutic ceiling of ICIs in upfront treatment of early-stage TNBC, elucidate the role of targeted therapies in increasing therapeutic index of ICIs, and identify reliable biomarkers to guide the imperfect prognostic value of PD-L1 expression.

Based on the remarkably durable responses in a small subset of TNBC responders in ICI monotherapy studies, many studies using combination ICI with conventional therapies are currently ongoing and have shown early signs of benefit. Interim data from Impassion130 trial, using nab-paclitaxel in combination with Atezolizumab showed a 40% ORR in metastatic TNBC, and early data suggests a clinically meaningful overall survival benefit in patients with PD-L1 immune cell-positive disease (NCT02425891). Trials investigating the combination of Eribulin and Pembrolizumab in heavily pretreated metastatic TNBC are ongoing, with interim analysis demonstrating a 41.2% ORR to first-line treatment and a 27.3% ORR to later-line treatment (107). However, PD-L1 status failed to predict treatment response to either combination. These trials investigating ICI efficacy in heavy-treated TNBC patients altogether have highlighted the need for earlier intervention with ICI therapy in advanced or metastatic TNBC. KEYNOTE-355, a phase III trial evaluating the combination of Pembrolizumab plus conventional chemotherapy compared with chemotherapy alone as first-line treatment in metastatic TNBC is ongoing (NCT02819518). The combination of Durvalumab and nab-paclitaxel followed by dose-dense conventional chemotherapy as well as the combination of Avelumab and an antibody to the immune modulator, 41BB, is under investigation in advanced solid tumors, including TNBC (NCT02489448).

In early TNBC, preliminary results from the neoadjuvant I-SPY 2 trial demonstrated that pCR rates increased from 22.3% to 62.4% by adding neoadjuvant Pembrolizumab to paclitaxel followed by anthracycline-based chemotherapy, which represents an approximately 40% improvement in pCR compared with standard chemotherapy alone (108). The KEYNOTE-173 trial also showed a remarkably increased pCR rate from 60% to 90% in high-risk patients by combining Pembrolizumab with paclitaxel or conventional chemotherapy (109). In the adjuvant setting, the SWOG1418 phase III trial is evaluating adjuvant monotherapy with Pembrolizumab after neoadjuvant chemotherapy followed by curative surgery. Another phase III trial for high-risk patients with early TNBC is investigating the addition of Avelumab after standard curative treatment including adjuvant chemotherapy (NCT02926196).

Clinical success using immune checkpoint inhibitors has led to the identification of additional checkpoints that mediate tumor immunosuppression, such as the lymphocyte-activation gene 3 (LAG3), T cell immunoglobulin and mucin-domain 3 containing-3 (TIM3), Siglec-15, indoleamine 2, 3-dioxygenase 1 (IDO1), and glucocorticoid-induced tumor necrosis factor receptor (GITR). Targeted therapies for these are undergoing clinical trials in TNBC patients. For example, Siglec-15 is an immune checkpoint that inhibits antigen-specific T cell responses, and is expressed, independent of PD-L1 status, on both tumor and tumor-infiltrating myeloid cells (110), and a mAb for Siglec-15 is currently being evaluated in a phase I/II study for advanced or metastatic solid tumors (NCT03665285).

Although clinical trials for immunotherapy in breast cancer have not shown that same high efficacy as in other carcinomas, TNBC is likely to have increased benefit as compared to other types of breast cancer given high mutational load, DDR-deficiency and increased PD-L1 expression. This may be especially true in early-stage TNBC with potentially more favorable tumor immune microenvironments, as studies thus far have mostly evaluated immune checkpoint inhibitors in advanced staged TNBC. However, innate, and adaptive resistance to immunotherapy remains a challenge, and targeted therapies that synergize with the immune-activating potential of immune checkpoint inhibitors is a promising strategy to maximize immunotherapeutic potential in TNBC patients.

## 3 DDR Deficiency-Associated Anti-Tumor Immunity in TNBC

Recent work has highlighted the important interaction between genomic instability and the immunogenicity and activation of anti-tumor immunity (111). Highly mutated tumors often exhibit one or several mutations in key components of DDR or replicative pathways, including MSH2 for MMR/MSI, BRCA1/2 for HR and DNA polymerase epsilon (POLE) for DNA replication. Targeting of DSB repair proteins with DDR inhibitors has also been shown to increase the TMB (111). Likewise, DDR defects result in accumulation of chromosomal abnormalities, leading to higher TMB, oncogene activation and tumorigenesis (112, 113). However, this DDR-defect-dependent genomic instability and increased TMB can also result in immune recognition, activation of immunostimulatory genes, increased TIL, and anti-tumor immune production of IFN-γ with resultant immunosuppressive tumor PD-L1 upregulation (18, 90, 114, 115). Similar effects can be observed as a result of genomic stress induced by DDR defects or DNA-damaging treatments, including RT, PARPi or platinum-based chemotherapies. This is due to generation of chromosomal fragments that stimulate the cytosolic sensing cGAS/STING pathway that promotes anti-tumor immunity through activation of T and NK cells, neoantigen recognition, and increased PD-L1 expression, and this immune system stimulation is enhanced in the background of BRCAness/HRDness (24, 27, 90, 116–118). It is also evident that in response to DNA damage, ATM/ATR/CHK1 kinase activity regulates the transition from DDR to immunostimulatory signaling directly through STAT1/3-IRF1-mediated transcription of PD-L1 (26). Thus, DDR signaling and DNA-damaging treatments result in robust immune modulation and significantly affect the balance between tumor progression and immune surveillance.

In support of the notion that tumor cells with extensive genomic instability orchestrate a high octane anti-tumor immune response that is smothered by coexisting immunosuppression, DNA damage and DDR-defects associated with increased TMB and neoantigen production correlate with STING-induced PD-L1 expression and improved ICI response (25). In some studies including in invasive breast carcinoma, defects in BER or BRCA1/2 were associated with increased neoantigen load, increased TILs, and elevated PD-L1 expression (26, 90, 91, 119), and a genome wide genetic screen identified BRCA2 inactivation as a mediator of cGAS/STING-induced IFN response and pro-inflammatory cytokine production (116). Consistent with these findings, DDR deficient breast tumors exhibited increased immune infiltration. However, elevated PD-L1 expression was driven predominantly by cGAS/STING pathway activation as opposed to the canonical neoantigen/activated T cell/IFN-γ pathway of PD-L1 induction (69), which is significant given that STING activation mediated by DNA-damaging agents is implicated in response to ICI therapy. Although some studies report elevated TILs in BRCA1/2 mutant breast cancer (21, 24), a pooled analysis of five phase II studies showed that TIL density was not associated with HR defect or BRCA1/2 mutation in early stage patients with TNBC (20). It is therefore likely that neoantigen-independent mechanisms of immune augmentation are involved in TIL density and PD-L1 expression in DDR-deficient TNBC, which is compatible with numerous studies that have shown tumors with low TMB can also be sensitive to ICIs (22). Importantly, patients with BRCA1/2 and other HR-related gene deficiencies demonstrate higher response rates to ICI as compared to MMR deficient tumors despite relative lower TMB, corroborating the possibility of additional immunologic mechanisms related to DDR-deficiency (22, 120, 121). Furthermore, the observation that HR intact tumors may also respond to the PARPi and ICI combination could perhaps be explained by the activation of the cGAS/STING and subsequent neoantigen-independent immune activation (122, 123).

Despite the significant clinical activity of PARPi in breast cancers harboring germline loss-of-function BRCA mutations (14, 16, 39), the majority of patients treated with PARPi’s alone do not significantly benefit (115). Unrepaired chromosomal damage following PARPi further promotes immune activation and adaptive upregulation PD-L1 expression *via* cGAS/STING pathway activation or ATM/ATR/CHK1 kinase activity (26, 119), which may result in immune escape and explain variable results. Altogether these studies support the hypothesis that use of PARPi together with ICI will retain immune activating consequences of DDR defect targeting while also preventing T cell inactivation.

In the setting of DDR deficiency, a consequence of tumor cell DNA damage and sustained inflammatory activity is recruitment and activation of immunosuppressive immune phenotypes as the result of chronic, low level, DNA damage, potentially resulting in cancer progression and immunotherapy resistance (117). It is proposed that PARPi may potentially shift to more substantial DDR-mediated cytotoxic anti-tumor immune milieu more favorable for ICI efficacy (124). In support, it is reported that PARPi efficacy is enhanced by CD8^+^ T cell activity *via* cross-talk with STING pathway activation in BRCA-deficient models of TNBC (123). Collectively, numerous studies indicate PARPi-dependent immunologic effects may prime a vigorous albeit imbalanced anti-tumor immune response and set the stage for improved ICI efficacy.

The combination of enhanced immune activation resulting from deficient DDR pathway signaling and the immunosuppressive consequences, including PD-L1 upregulation of unrepaired DNA damage *via* HR-deficiency and/or the use of DDR inhibitors such as PARPi, suggests potentially targetable immunological susceptibilities in TNBC patients (**Figure 2**). Tumor immune evasion mechanisms in response to genomic instability subvert immune-mediated elimination of DDR hindered cancers, serving as rationale for targeting the immunosuppressive arm of DDR signaling in response to DNA damaging therapies *via* ICI combinations. This approach may be highly lethal to immunogenic tumor cells with DDR defects and impinge upon these immunosuppressive mechanisms of therapeutic resistance (24, 68, 71, 125, 126). TNBC often harnesses DDR defects, TMB load, and PD-L1 expression, and these characteristics have been found to be amongst the strongest predictors of response to ICI (18, 22, 113).

### 3.1 DDR Inhibitors and Immunotherapy in TNBC

Therapeutic targeting of genomic instability through the use of DDR-inhibitors, including PARPi, have been shown to not only induce synthetic lethality in DDR-deficient tumor cells, but also to augment the tumor immune microenvironment through increased TMB and activation of immunostimulatory genes (21, 25, 114). Accruing evidence supports the potential association between DDR defects and ICI efficacy. Interestingly, preclinical TNBC studies demonstrated PARPi-mediated PD-L1 upregulation with expected attenuation of anti-tumor immunity, that PD-L1 blockade re-sensitized PARPi-treated cancer cells to T-cell killing, and the combination of PARPi and anti-PD-L1 therapy demonstrated greater antitumor activity and tumor control compared with each agent alone (127), further indicating a potential synergistic effect of combination DNA damage response inhibitors (DDRi’s) and ICI. Combination PARPi with PD-1/PD-L1 targeted therapies demonstrated increased TILs and enhanced antitumor immunity in both BRCA-proficient and BRCA-deficient mouse models of TNBC (123, 127), indicating additional PARPi-mediated immunologic factors associated with ICI outcomes. Interestingly, whole exome sequencing of cancer patients previously treated with PD-1 inhibitors revealed that ICI responders are enriched for BRCA mutations (8). Altogether, this dual effect of DDRi-induced immune activation and PD-L1-dependent immunosuppression suggests immunologic vulnerability that may be exploited through the use of ICI, and serves as the rationale for studies investigating the clinical efficacy of combination therapy with PARPi’s and anti-PD-1/PD-L1 in multiple cancers, including TNBC (121, 128).

A summary of ongoing clinical trials combining DDR targeting agents with immunotherapy is listed in **Table 1**. 

**Table 1:** Ongoing clinical trials of combination DNA targeting and/or immunotherapy agents in TNBC or BC with DDR mutations.

Phase	Trial ID	BC subtype	Biomarkers	Regimen	Targets	Clinical endpoint
**I**	NCT03544125	mTNBC	Pre- and post-tumor biopsy (CLIA) analytics	Olaparib + Durvalumab	PARP PD-L1	Safety, ORR, DOR, PFS, OS
**I**	NCT03101280	Advanced or mTNBC	–	Rucaparib + Atezolizumab	PARP PD-L1	DLTs, PK, ORR, CR, PFS
**I/II**	NCT03964532 TALAVE	Advanced BC	Germline BRCA1/2 Deleterious mutation OR BRCA1/2 wild status TNBC; Serial biopsies for PD-L1	Talazoparib + Avelumab	PARP PD-L1	Safety, ORR, PFS, OS
**II**	NCT04584255	BRCAm Stage I-III BC	BRCA mutations, pre- and post-TILs, STING activation, serum immune	Niraparib + Dostarlimab	PARP PD-1	pCR, RCB
**II**	NCT02849496	HER- mBC	BRCA 1/2 mutation, HRD, PD-L1, TILs, ctDNA	Olaparib + Atezolizumab	PARP PD-L1	PFS, TTF, ORR, DOR, irBOR
**II**	NCT03801369	mTNBC	Tumor characteristics, predictive biomarkers	Olaparib + Durvalumab	PARP PD-L1	ORR, OS
**II**	NCT03025035	Advanced BRCAm BC	germline mutations in BRCA1 or BRCA2	Olaparib + Pembrolizumab	PARP PD-1	ORR, PFS, OS, irRECIST
**II**	NCT03167619 DORA	Advanced or mTNBC	Molecular biomarkers, TILs, PD-L1 status, cTC, plasma DNA	Olaparib + Durvalumab	PARP PD-L1	PFS, CR, PR, SD, OS
**II/III**	NCT04191135 KEYLYNK-009	Advanced TNBC	-	Olaparib + Pembrolizumab	PARP PD-1	PFS, OS
**I/II**	NCT03594396 MEDIOLA	Stage II/III TNBC	Serial tumor and serum biopsy study	Olaparib + Durvalumab	PARP PD-L1	pCR, ORR
**I/II**	NCT02484404	Advanced or mTNBC	gBRCAm status	Olaparib + Durvalumab	PARP PD-L1	Safety, ORR, PFS
**I/II**	NCT02657889 TOPACIO	Advanced or mTNBC	–	Niraparib + Pembrolizumab	PARP PD-1	DLTs, ORR, DOR, PFS, OS, PK
**II**	NCT04169841 GUIDE2REPAIR	HR-mutated advanced or metastatic BC	HR repair gene mutations	Olaparib + Durvalumab + Tremelimumab	PARP PD-L1 CTLA-4	Safety, PFS
**II**	NCT03330847	mTNBC	BRCA1/2 mutations or HRRm	Olaparib + Ceralasertib or Adavosertib	PARP ATR WEE1	PFS, ORR, OS, DOR, PK
**I**	NCT03945604	Advanced or mTNBC	-	Apatinib + Fluzoparib + Camrelizumab	VEGF PARP PD-1	DLT, ORR, PFS, OS
**II**	NCT04837209 NADiR	mTNBC	TILs, ctDNA	Niraparib + Dostarlimab + RT	PARP PD-1 DNAx	ORR, irRECIST, OS, PFS
**I/II**	NCT02264678	Her2- BC with BRCAm or TNBC	BRCA mutations HRRm ATR inhibition, ctDNA, CTCs	Ceralasertib + Durvalumab	ATR PD-L1 DNAx	Safety, PK, ORR, PFS, OS
**I**	NCT01618357	Stage II-IV BC, residual after NAC	Apoptosis/proliferation biomarkers	Pre-operative Veliparib + RT	PARP DNAx	Safety, MTD
**I**	NCT03945721 UNITY	Non-mTNBC	HRD status	Niraparib + post-op RT	PARP DNAx	MTD, LRR, DFS, cosmesis
**I**	NCT02227082	Advanced or mTNBC	–	Olaparib + RT	PARP DNAx	Toxicity
**I**	NCT03542175	Post-op TNBC	-	Rucaparib + RT	PARP DNAx	MTD
**I**	NCT04052555	Non-mTNBC	DDR mutations	Berzosertib + RT	ATR DNAx	MTD, DFS, OS
**I**	NCT02977468 Pembro/IORT	Treatment naïve TNBC	TILs	Pembrolizumab + intra-op RT	PD-L1 DNAx	-
**II**	NCT03464942 AZTEC	Advanced TNBC	–	Atezolizumab + stereotactic RT	PD-L1 DNAx	PFS, ORR, DOR, OS
**I**	NCT02826434	Stage II/III TNBC, HLA-A2+	Immune response rate, vaccine-specific CTLs	Peptide vaccine + Durvalumab	XBP1,CD138 PD-L1	Safety, tolerability

mTNBC, Metastatic triple-negative breast cancer; BC, breast cancer; (CLIA) analytics, Proportion of completion of Clinical Laboratory Improvement Act; ORR, objective response rate; DOR, duration of response; PFS, progression-free survival; OS, overall survival; DLTs, Dose-Limiting Toxicities; MTD, maximum tolerated dose; PK, Pharmacokinetics; pCR, pathologic complete response; TILs, tumor infiltrating lymphocytes; STING, stimulator of interferon genes; RCB, pathway, residual cancer burden; HRD, homologous recombination deficiencies; HRRm, HRR-related gene mutation; ctDNA, circulating tumor DNA; TTF, time to treatment failure; irBOR, immune-related best overall response; irRECIST, immune-related; cTC, circulating tumor cells; SD, stable disease; gBRCAm, germline BRCA1 and BRCA2 mutation; DDR, DNA-damage response; DCR, pathway, disease control rate; LRR, locoregional relapse; DFS, distant relapse; CTLs, Cytotoxic T Lymphocytes; DNAx, therapeutic targeting of DNA DSBs; (HLA)-A2+, Human Leukocyte Antigen.

### 3.2 Exploiting BRCA1/2 Deficiency and Immunotherapy in TNBC

Given the potential of tumor cell HR defects, including BRCAness, to increase susceptibility to ICI through enhanced immune activation and expression of PD-1 or PD-L1 (103), ICI response is being studied in cancers, including breast cancers, with germline mutations in BRCA1 or BRCA2 (NCT01772004, NCT03025035). In previously treated, platinum-resistant recurrent ovarian cancer, Durvalumab and Olaparib demonstrated clinical activity, irrespective of BRCA mutation status (NCT02484404) (129). Interestingly, analysis of core biopsy and blood samples revealed this combination created a stronger immunostimulatory phenotype with enhanced IFN-γ and CXCL9/CXCL10 expression, systemic IFN-γ/TNF-α production and TILs (128, 129). Combination treatment with Durvalumab with the PARPi Olaparib is currently under exploration in a phase I/II trial of women’s cancers, including patients with TNBC, with biomarker evaluation ongoing (128). The phase II MEDIOLA basket trial assessed the efficacy and safety of combination Olaparib and Durvalumab in patients with solid tumors, including ovarian cancer, breast cancer and gastric cancer (NCT02734004). In germline BRCA mutant, platinum-sensitive relapsed ovarian cancer, this combination demonstrated an overall response rate (ORR) of 63% and a 12-week disease control rate (DCR) of 81% (15). In gBRCAm HER2 negative metastatic breast cancer, the DCR was 80% at 12 weeks and 50% at 28 weeks, with ORR of 63%. Median PFS (mPFS) was 9.2 months and median overall survival (mOS) was 21.5 months. Moreover, patients with no prior line of chemotherapy had higher ORR and longer OS than those with two prior lines (respectively 78% *vs.* 50% for ORR and 21.3 *vs.* 16.9 months for OS) (15). Although there is no observed association between PD-L1 positivity and TILs at this point in the trial, there was a trend of higher PD-L1 and increased TILs observed in archival samples in patients who had SD/PR/CR, which was not observed in patients with progressive disease. Furthermore, high PD-L1 was observed in patients with DCR at 12 weeks (15, 33). In the phase II TOPACIO trial (NCT02657889), Niraparib and Pembrolizumab combination therapy has demonstrated clinical benefit in platinum-resistant TNBC, with numerically higher response rates in those with BRCA-mutated TNBC tumors (ORR of BRCAm *vs.* BRCA wild-type, 47% *vs.* 11%) (130). A phase II multicenter study of Durvalumab and Olaparib is underway for patients with advanced TNBC that is inoperable, locally advanced, or metastatic, and is not amenable to resection with curative intent, and who have received at least 4 cycles of platinum-based chemotherapy with demonstrated clinical benefit (NCT03167619). Other trials combining PARPi’s, including Olaparib, Rucaparib, and Fluzoparib, with ICIs, such as Pembrolizumab (NCT03101280), Atezolizumab (NCT04191135), and Camrelizumab (NCT03945604), respectively, for locally advanced or metastatic TNBC are also underway. A phase II study will evaluate safety and efficacy of combination of PARPi (niraparib), PD-1 mAb (Dostarlimab), and RT in metastatic TNBC (NCT04837209). Though the relationship between endogenous or PARPi-induced BRCAness and immunotherapy response is still being investigated, these ongoing clinical trials will help establish the effect HR-deficiency and DDR targeting therapies on ICI outcomes in TNBC.

### 3.3 Other DDR Targets and Immunotherapy in TNBC

Evidence that unrepaired DNA damage induced by PARPi expands the anti-tumor activity of the ICI has prompted investigation of other key mediators implied in DNA replication and repair, such as ATM, ATR, CHK1, CHK2, DNA-PK, and WEE1 (31, 120, 121). Given the immunomodulatory effects seen with PARPi, these additional DDR mediators are exciting targets for combined immunotherapy. In preclinical breast cancer studies, the combination of a selective ATR inhibitor with Avelumab and platinum-based chemotherapy resulted in antitumor effect in syngeneic tumor models, leading to overall survival benefit compared to any dual-combination group, and also provided protective antitumor immunity with immunological memory in cured mice (131). In a preclinical model of lung cancer, CHK1 inhibition potentiated the anti-tumor effect of PD-L1 blockade and augmented cytotoxic T cell infiltration (27). In other studies, inhibition of DNA-PK upregulated PD-L1 in a cGAS-STING-dependent manner in irradiated p53-mutant cancer cells, suggesting selective blockade of NHEJ repair of DSB exhibits immunomodulatory effects similar to those seen in HR-inhibition. Preclinical studies of combined DNA-PK inhibition, radiation and PD-L1 blockade demonstrated increased anti-tumor activity in a p53-mutant cancer, suggesting that inhibition of DNA-PK inhibits repair of radiation-induced DSBs resulting in potentiation of anti-tumor immunity, adaptive PD-L1 expression through DDR-dependent mechanisms, and subsequent responsiveness to immune checkpoint blockade (132).

These promising preclinical studies have led to several early phase clinical trials. A clinical study in patients with advanced or metastatic cancers, use of Ceralasertib, a potent and selective ATR inhibitor in combination with Durvalumab is being evaluated (NCT02264678). A selective ATR kinase inhibitor, AZD6738, is undergoing a phase II study with Olaparib for metastatic TNBC patients with BRCA1/2 mutations or HRD (NCT03330847). The phase Ib BISCAY study, Durvalumab and Olaparib or the WEE1 inhibitor Adavosertib in patients with metastatic cancer with any detected HR-deficiency (NCT02546661). A phase I study combining the CHK1 inhibitor, Prexasertib, with a PD-L1 mAb demonstrated the potential for enhanced therapeutic activity and increased cytotoxic T cell activation (125).

Further highlighting the indication that DDR-inhibitors and DNA-damaging agents may enhance immunotherapeutic response, a phase II clinical trial is evaluating the efficacy of Atezolizumab with stereotactic RT for advanced TNBC (NCT03464942), and a phase I study for the feasibility of adjuvant Durvalumab with a peptide vaccine is underway for patients with stage II and III TNBC after completion of standard adjuvant therapy (NCT02826434). Altogether, early studies indicate a potential therapeutic benefit of DDR-pathway targeting/inhibition in combination with immunotherapy, and ongoing trials will provide new insights into and establish clinical efficacy of the immune potentiating efficacy of DDR-inhibitors.

## 4 Summary

TNBC represents a highly diverse set of breast cancers with complicated molecular and immunologic landscapes, and thus remains a challenging oncologic entity to tackle effectively. However, advances in genomic profiling and our understanding of the interplay between DNA damage response and cancer immunity has resulted in exciting immuno-molecular therapeutic opportunities. Of these, DDR-deficiencies including BRCAness have been shown to promote immunologic vulnerability through DNA damage-induced high TMB, immune-stimulatory and suppressive features, as well as adaptive immune resistance *via* PD-L1 upregulation. DDR deficiencies represent a frequent aberration in TNBC, and exploitation of immunologic consequences offers potential therapeutic leverage that combines favorable immune effects of DNA/DDR-targeted therapies with restoration of cytotoxic anti-tumor immune cells. The role for endogenous as well as therapy-induced DNA damage signaling in PD-L1 induced expression, and the possibility of circumventing DNA targeted therapy-induced immune suppression with concomitant immunotherapy provide rationale for combining agents targeting the DDR and the immune system. Immunotherapy, chiefly ICI, represents an opportunity to flip the switch back to immune activation, particularly in the context of concomitant DDR pathway targeting therapies, such as PARP inhibitors and others.

PARP inhibitor monotherapies, as well as therapeutic combinations, have demonstrated promising clinical benefit, and their effects on enhancing lethal DNA damage vulnerabilities have been shown. Nonetheless, the underlying mechanisms of PARPi-mediated sensitization of tumors to immunotherapy and/or radiotherapy remain to be fully elucidated. Furthermore, rapid translation of these potential breakthroughs in TNBC treatment will require thoughtful incorporation and thorough dissection of clinical trial outcomes and their implications into everyday clinical practice. Despite preclinical and clinical studies that have demonstrated PARPi-mediated immunosuppression *via* PD-L1 induction and complementary restoration of PARPi sensitivity *via* PD-L1 inhibition, with the added possibility of enhanced anti-tumor immunity, many unanswered questions remain regarding the potential benefit of combined targeted therapies and ICIs in TNBC. In addition to PARPi, other repair pathway mediators such as ATR, and CHK1, are being investigated in combination with immune-based strategies, and thus careful consideration of promising therapeutics as well as other immunotherapeutic strategies in the pipeline should not be overlooked. Optimization of treatment schemas for combined immunotherapeutic strategies remains a challenge, as does validation of biomarkers that will identify which patients will most benefit from either PARP inhibitors in combination with immunotherapy, radiotherapy, or other targeted therapies.

Lastly, identifying additional key mediators of DNA damage-associated immune modulation that regulate disease progression, therapeutic response and resistance will require further preclinical investigation and careful analysis of clinical samples to assess DDR deficiencies in certain tumor subsets, with the ultimate goal of personalizing DNA targeting and immune-based therapies in combination with conventional DNA- and immune-augmenting therapies, such as chemotherapy and radiation, to maximize the combined benefits of each approach and effectively target immunosuppressive pathways that contribute to immune escape and tumor progression. It will also be important to identify mediators of poor response to ICIs and improved prognostic markers for existing therapies to select patients that may benefit from alternative therapeutic strategies and explore options for TNBC refractory to ICI or PD-L1 negative TNBC. Furthermore, the role of less studied DDR mechanisms related to ICI is still unclear, and future work is needed to better predict which DNA damage response and repair pathways are most suitable for therapeutic targeting in specific subsets of patients.

## Author Contributions

Both authors initiated the concept and wrote the manuscript. All authors contributed to the article and approved the submitted version.

## Funding

This work was supported by the UAB Department of Radiation Oncology.

## Conflict of Interest

EY: Advisory Board: Astrazeneca, Bayer, Clovis, Strata Oncology; Research Support to Institution: Eli Lilly, Novartis, Puma Biotechnologies.

The remaining author declares that the research was conducted in the absence of any commercial or financial relationships that could be construed as a potential conflict of interest.

## Publisher’s Note

All claims expressed in this article are solely those of the authors and do not necessarily represent those of their affiliated organizations, or those of the publisher, the editors and the reviewers. Any product that may be evaluated in this article, or claim that may be made by its manufacturer, is not guaranteed or endorsed by the publisher.
